# Determining Methyl-Esterification Patterns in Plant-Derived Homogalacturonan Pectins

**DOI:** 10.3389/fnut.2022.925050

**Published:** 2022-07-01

**Authors:** Yang Yu, Liangnan Cui, Xianbin Liu, Yuwen Wang, Chenchen Song, UnHak Pak, Kevin H. Mayo, Lin Sun, Yifa Zhou

**Affiliations:** ^1^Jilin Provincial Key Laboratory on Chemistry and Biology of Changbai Mountain Natural Drugs, Engineering Research Center of Glycoconjugates of Ministry of Education, School of Life Sciences, Northeast Normal University, Changchun, China; ^2^Department of Biochemistry, Molecular Biology and Biophysics, The University of Minnesota, Minneapolis, MN, United States

**Keywords:** HG pectin, endo-polygalacturonase, enzymatic fingerprinting, methyl-esterification, oligogalacturonides

## Abstract

Homogalacturonan (HG)-type pectins are nutrient components in plants and are widely used in the food industry. The methyl-esterification pattern is a crucial structural parameter used to assess HG pectins in terms of their nutraceutical activity. To better understand the methyl-esterification pattern of natural HG pectins from different plants, we purified twenty HG pectin-rich fractions from twelve plants and classified them by their monosaccharide composition, Fourier transform-infrared spectroscopy (FT-IR) signatures, and NMR analysis. FT-IR shows that these HG pectins are all minimally esterified, with the degree of methyl-esterification (DM) being 5 to 40%. To examine their methyl-esterification pattern by enzymatic fingerprinting, we hydrolyzed the HG pectins using endo-polygalacturonase. Hydrolyzed oligomers were derivatized with 2-aminobenzamide and subjected to liquid chromatography-fluorescence-tandem mass spectrometry (HILIC-FLR-MS^n^). Twenty-one types of mono-/oligo-galacturonides having DP values of 1–10 were found to contain nonesterified monomers, dimers, and trimers, as well as oligomers with 1 to 6 methyl-ester groups. In these oligo-galacturonides, MS^n^ analysis demonstrated that the number of methyl-ester groups in the continuous sequence was 2 to 5. Mono- and di-esterified oligomers had higher percentages in total methyl-esterified groups, suggesting that these are a random methyl-esterification pattern in these HG pectins. Our study analyzes the characteristics of the methyl-esterification pattern in naturally occurring plant-derived HG pectins and findings that will be useful for further studying HG structure-function relationships.

## Introduction

Pectin is a family of covalently linked, galacturonic-rich acidic polysaccharides widely found in the cell walls of plants. These are usually divided into homogalacturonan (HG), rhamnogalacturonan I (RGI), and rhamnogalacturonan II (RGII) domains (1). HG is the most abundant pectin, accounting for more than 65% of the total pectin (1). HG is a linear homopolymer that is primarily composed of α-1,4-linked D-galacturonic acid (GalA) residues with DP values of 30–100 (2), although shorter chains have also been reported (3). GalA residues in HG pectins can be methyl-esterified at their C-6 carboxyl group (4), with their degree of methyl-esterification (DM) and methyl-ester distribution being major structural characteristics of HG pectins. HG pectin is synthesized in the Golgi of plants and transported to the cell wall by vesicles (5). During synthesis, HG pectins can be modified by HG-methyltransferase (HG-MT) to form highly esterified pectin (6, 7), which can be de-esterified by pectin-methylesterases (PMEs) (8, 9). The methyl-esterification pattern in HG pectins is involved in normal physiology, as well as in plant pathology, e.g., in regulating growth (10), morphology, development (11), and defense (12, 13).

Homogalacturonan pectin, a crucial dietary nutrient in plants, has anti-inflammatory properties, as well as the ability to modulate immunity and intestinal flora. Due to this, HG pectin is regarded as a key functional factor in healthy foods that improve intestine health and metabolism (14). Many properties and nutraceutical functions of HG pectins are related to their DM values (15). Compared with high DM pectin, low DM pectin shows better antioxidant, anti-inflammatory, and immunomodulatory properties and is more conducive to regulating intestinal flora (14). Recently, it was reported that not only the DM value but also methyl-ester distribution can influence anti-inflammatory and immunoregulation properties of pectin. Low DM pectins, as well as intermediate DM pectins with blockwise distributions of nonesterified GalA residues, are beneficial for anti-inflammatory effects *via* inhibition of TLR2-1 receptors (16, 17). In addition, low DM pectins with a higher blockwise distribution of nonesterified GalA residues, as well as intermediate DM pectins, have been confirmed to increase the frequency of intestinal T-helper (Th)1 and Th2 cells in mice (18). Therefore, it is necessary to analyze methyl-esterification patterns in HG pectins to further investigate the relationship between their structural features and their nutritional functions.

Due to the high molecular weight of HG pectins, it is difficult to directly assess methyl-esterification patterns. Enzymes such as endo-polygalacturonase (Endo-PG), endo-polygalacturonase (PL), and pectin methyl esterase (PME) (19, 20) have been used to hydrolyze and modify HG pectins for structural analyses. HG-related oligomers produced by enzyme hydrolysis have been separated and analyzed using high-performance anion-exchange chromatography (HPAEC at pH 5) and capillary electrophoresis (21, 22). Nevertheless, the structural characteristics of these oligogalacturonides remain unknown. In recent years, hydrophilic interaction liquid chromatography (HILIC), coupled with electrospray ionization ion trap mass spectrometry (ESI-MS^n^), has been employed in the analysis of oligosaccharides (23–25), an approach that can be used to separate oligogalacturonides and analyze their structures simultaneously. However, it has been difficult to distinguish B/C and Y/Z fragment ions of these oligosaccharides in HILIC-ESI-MS^n^, thus leading to inaccurate structural results (26).

Derivatization is often used prior to MS analysis of oligosaccharides, e.g., by using reductive amination to add a fluorescent probe at the reducing end of the oligosaccharides (27, 28). In this approach, the fluorescence detector only observes oligosaccharides that have fluorescent markers, whereas impurities will not be detected (29). Oligosaccharide isomers with different chiralities are excluded, as the reducing end is labeled, making the chromatogram more easy to analyze. In this regard, a variety of fluorescent probes have been used, including 2-aminobenzamide (2-AB), 2-aminobenzoic acid (2-AA), 2-aminopyridine (2-AP), and 2-aminoacridone (AMAC) (29–32). HG methyl-ester distribution can be determined by analyzing the structural characteristics and quantifying oligogalacturonides produced.

To characterize the methyl-esterification pattern in HG pectins, we used a series of descriptive parameters, including the degree of blockness (DB) and the absolute degree of blockiness (DB_abs_) (20, 24, 33). DB values were calculated as the amount of nonesterified monogalacturonic, digalacturonic, and trigalacturonic acid residues released by endo-PG relative to the total unesterified GalA (20). However, determining the DB value may be complicated by the DM value of a pectin, especially those with high DM values. Due to this, we used DB_abs_ values to provide information about the absolute number of blocks in the pectin without correction for DM and values that are calculated as the number of unesterified GalA residues in enzymatic oligogalacturonides relative to the total GalA (33). Both DB and DB_abs_ have been commonly used to characterize random and blockwise patterns of methyl-esterification in HG pectins (24). PL has also been used to degrade HG pectins in order to study their methyl-esterification pattern in highly esterified pectins, as well as the degree of blockiness and the absolute degree of blockiness in highly methylesterified stretches (DB_Me_ and DB_absMe_, respectively) (20). Using the combined degradation of two glycosidases to study the methyl ester distribution in HG pectins has also been reported in recent years, and the concept of the pectin degree of hydrolysis (DH) has been proposed to describe methyl-esterification patterns (24).

Recently, we have been carrying out a program about comparing pectin structures of plants, which are used as food or herbs in China (34, 35). In this study, we reported the results of twenty HG pectins purified from twelve plants first. These twenty HG pectins were hydrolyzed using Endo-PG, and the resulting HG oligomers were derivatized with 2-AB and analyzed using HILIC-FLR-MS^n^. DB and DB_abs_ parameters were calculated and used to assess the distribution of methyl-ester groups. Our study reveals the characteristics of methyl-esterification patterns from natural HG pectins from plants and findings that will provide a basis for studying their structure-function relationships and formulating the use of HG pectins in healthy foods.

## Materials and Methods

### Materials and Reagents

DEAE-cellulose, Sepharose CL-6B, Sephadex G25, and aminobenzamide (2AB) were purchased from Sigma-Aldrich. Endo-polygalacturonase (Endo-PG, EC 3.2.1.15 from *Aspergillus niger*) was purchased from Megazyme. All chemicals used were analytical grade and produced in China.

Homogalacturonan pectins were extracted from the following plants: *Panax japonicus*, *Pseudostellaria heterophylla*, *Schisandra chinensis*, *Prunella vulgaris*, *Panax Notoginseng*, *Polygonum orientale*, *Anemarrhena asphodeloides*, *Kadsura longipedunculata*, *Isatis indigotica*, *Aconitum carmichaelii*, *Coptis chinensis*, and *Sophora flavescens*.

### Preparation of HG Pectins

The total polysaccharide was extracted from dried plants (MP) as previously described (34) and fractioned using DEAE cellulose ion-exchange chromatography with distilled water and 0.5 M NaCl as eluents to obtain neutral (MPP-N) and acidic polysaccharides (MPP-A). Total pectin extracts were loaded onto a DEAE cellulose column and eluted using a stepwise gradient of aqueous NaCl (0, 0.2, and 0.3 M) to acquire a charge distribution of homogenous fractions. These fractions were further purified using a Sepharose CL-6B column to obtain HG pectins (HG-MP). Elution curves were monitored by determining the sugar content.

### General Methods

Total carbohydrate content was determined using the phenol-sulfuric acid method (36). UV spectra are shown in Supplementary Methods 1. The molecular weight distribution was determined as shown in Supplementary Methods 2. Monosaccharide composition is shown in Supplementary Methods 3. ^13^C NMR spectra were obtained and are shown in Supplementary Methods 4.

### Degree of Polymerization

The degree of polymerization was analyzed using HPSEC-RI-MALLS. This system is comprised of one column (Shodex OH-Pack SB-803 HQ) connected to a multi-angle light scattering detector (DAWN HELEOSΠ, Wyatt Technology Corp., Santa Barbara, CA, United States) and a refractive index detector (OptilabrEX, Wyatt Technology Corp). The eluent was a solution of 0.2 M NaCl containing 0.02% NaN_3_. MWs were calculated using Astra (Version 6.1.1.84) software. Finally, the degree of polymerization was estimated by MW.

### Estimation of the Degree of Methyl-Esterification

The degree of methyl-esterification (DM) was calculated using Fourier transform-infrared spectroscopy (FT-IR) (Perkin Elmer, United States) spectroscopy as previously described (37). DM values are proportional to the ratio of the area from the band at 1,740 cm^–1^ over the sum of the areas of bands at 1,740 and 1,630 cm^–1^. To quantify the DM of samples, a calibration curve was constructed based on pectin standards of known DM (0, 22, 44, 66, and 88%).

### Enzymatic Hydrolysis

Homogalacturonan pectins (5 mg ml^–1^) were solubilized in 50 mM ammonium acetate buffer (pH 4.5). Notably, 1 μl of endo-polygalacturonase II (1,100 U ml^–1^) was added prior to incubation for 12 h at 40°C, and enzymatic hydrolysis was repeated once. The enzyme was inactivated by boiling at 100 °C for 10 min. After cooling, digests were centrifuged at 12,000 rpm for 5 min, and the supernatant was analyzed for the molecular weight distribution using high-performance size-exclusion chromatography. Oligo-galacturonides were purified on a Sepahdex-G25 column.

### Derivatization of Oligogalacturonides With Aminobenzamide (2-AB)

Oligogalacturonides were lyophilized and reconstituted with 10 μl of a 0.1 M 2-AB solution in glacial acetic acid/dimethyl sulfoxide (DMSO) (3:7, v v^–1^) and 10 μl of a freshly prepared solution of 1 M sodium cyanoborohydride in glacial acetic acid/dimethyl sulfoxide (DMSO) (3:7, v v^–1^). Solutions were centrifuged at 12,000 rpm for 1 min at room temperature. The reaction mixture was then incubated at 65°C for 3 h and concentrated to 200 ng μl^–1^ using 50% acetonitrile-water at a 1:1 ratio (vol vol^–1^) prior to analysis using HILIC-FLR/ESI-MS^n^ (32).

### HILIC-FLR/ESI-MS^n^ Analysis

Fluorescently labeled galacturonate-based oligosaccharides were analyzed using a UPLC system (Waters Acquity H-Class, United States) coupled to a fluorescence detector (Waters Acquity H-Class, United States) and an ESI-IT-MS^n^-detector (Amazon speed ETD, Bruker, Germany). Chromatographic separation was performed on an Acquity UPLC BEH amide column (1.7 m, 2.1 mm × 150 mm) in combination with a Van Guard pre-column (1.7 m, 2.1 mm × 5 mm; Waters Corporation, Milford, MA, United States). HILIC-FLR/ESI-MS^n^ elution procedures and detection methods are described in Supplementary Methods 5. Quantification of oligogalacturonides was verified using this procedure as illustrated in Supplemental Methods 6.

### Determination of the Degree of Blocked Segments and the Absolute Degree of Blocked Segments

The calculation of two parameters, namely, degree of blocked segments (DB) and absolute degree of blocked segments (DB_abs_) was determined using HILIC-FLR-MSn for quantification with 20 types of HG pectins. As previously described, DB and DB_abs_ were calculated as the number of unesterified GalA residues in oligogalacturonides relative to the total unesterified GalA and all the GalA residues in the polymer. These parameters were used to characterize the random or blocked patterns of methyl-esterification in HG pectins (18, 22, 31). The calculation formula is shown as follows:


(1) $$D\,B=\frac{\sum_{\text{n}=1-3}{\left[O\,l\,i\,g\,o\,G\,a\,l\,A_{\text{n}}\right]}_{u\,n\,e\,s\,t\,e\,r\,i\,f\,i\,e\,d}\times \text{n}}{G\,a\,l\,A_{u\,n\,e\,s\,t\,e\,r\,i\,f\,i\,e\,d}}$$

(2) $$D\,B_{a\,b\,s}=\frac{\sum_{\text{n}=1-3}{\left[O\,l\,i\,g\,o\,G\,a\,l\,A_{\text{n}}\right]}_{u\,n\,e\,s\,t\,e\,r\,i\,f\,i\,e\,d}\times \text{n}}{G\,a\,l\,A_{t\,o\,t\,a\,l}}$$

## Results and Discussion

### Preparation of HG Pectins From Plants

**FIGURE 1:**
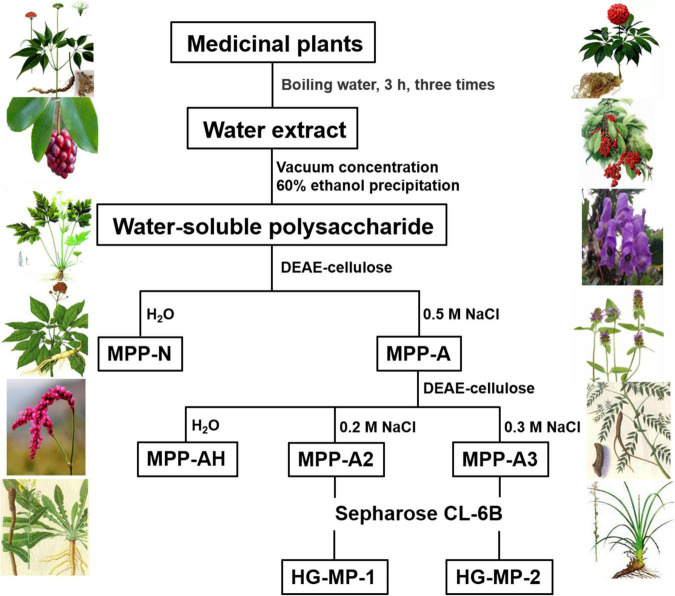
Separation and purification of homogalacturonan (HG) pectins from plants.

Homogalacturonan pectins were prepared from 20 plants according to the protocol as shown in Figure 1. In brief, total polysaccharide was obtained from each plant by hot water extraction and ethanol precipitation. Neutral (MPP-N) and acidic polysaccharides (MPP-A) were separated from total polysaccharides by ion-exchange chromatography. The acidic polysaccharides from different plants were all pectins, which were further separated by ion-exchange chromatography using different elution concentrations of sodium chloride solution. For some plants (*Isatis indigotica, Aconitum carmichaelii, Coptis chinensis*, and *Sophora flavescens*), MPP-A2 was the dominant fraction, whereas for other plants, both MPP-A2 and MPP-A3 were the major fractions. MPP-A2 from four plants and MPP-A2 and MPP-A3 from eight plants were then purified by size exclusion chromatography, and one major acidic fraction was obtained from each of them. Therefore, twenty pectin fractions came from twelve plants. Those fractions with relatively high yields and with homogenous molecular weight distributions (Supplementary Figure 1) were selected for further analysis. In UV-Vis spectra of all purified pectin fractions, no UV absorption was observed at 260 or 280 nm, indicating that they were free of proteins and nucleic acids (Supplementary Figure 2). The sugar content in these pectins ranged from 86.3 to 92.6%, indicating relatively high purity. Monosaccharide composition analysis showed that the GalA content in these fractions was over 70%, showing that HG was the major component in them. HPSEC-RI-MALLS analysis indicated that their weight-average molecular weights (MWs) ranged between 8 and 55 kDa (Supplementary Figure 1 and Table 1).

**TABLE 1:** Basic physicochemical properties of twenty kinds of homogalacturonan (HG) pectin.

Plant	Fraction*^a^*	Sugar content (% w)	Mw (KDa)	Monosaccharide composition (mol %)			
				GalA	Rha	Gal	Ara
*Panax japonicus*	HG-PJ-1	89.4	13.4	83.9	3.2	5.8	5.1
	HG-PJ-2	90.1	18.5	91.0	2.6	2.5	2.4
*Pseudostellaria eterophylla*	HG-PH-1	87.3	18.7	80.6	3.8	7.0	8.6
	HG-PH-2	86.4	26.0	81.1	4.2	6.2	8.5
*Schisandra chinensis*	HG-SC-1	91.5	51.0	90.8	2.5	3.3	1.9
	HG-SC-2	88.6	13.0	81.9	3.8	6.4	5.8
*Prunella vulgaris*	HG-PV-1	92.5	11.3	80.4	5.3	8.2	6.1
	HG-PV-2	90.6	21.0	78.1	8.9	7.4	5.6
*Panax Notoginseng*	HG-PN -1	91.6	11.8	74.6	5.1	9.3	8.0
	HG-PN-2	89.4	18.7	75.4	6.5	8.3	8.2
*Polygonum orientale*	HG-PO-1	86.3	26.9	75.9	9.4	8.7	6.0
	HG-PO-2	87.2	23.0	74.0	9.4	10.1	6.5
*Anemarrhena sphodeloides*	HG-AA-1	88.9	10.1	81.7	3.4	4.8	10.2
	HG-AA-2	91.2	15.5	74.6	5.9	4.9	14.6
*Kadsura longipedunculata*	HG-KL-1	92.6	19.1	81.1	4.2	7.5	7.2
	HG-KL-2	91.5	27.9	73.6	9.6	10.9	5.9
*Isatis indigotica*	HG-II	89.1	35.7	72.2	11.6	6.1	10.1
*Aconitum carmichaelii*	HG-AC	87.6	43.4	78.3	10.0	5.0	6.7
*Coptis chinensis*	HG-CC	88.0	16.1	70.6	4.8	7.4	17.2
*Sophora flavescens*	HG-SF	89.1	48.0	71.2	7.6	7.2	13.9

*^a^The pectin fractions were named “HG-binomial nomenclature of plants (initials).”*

### ^13^C-NMR Analysis of HG Pectins

**FIGURE 2:**
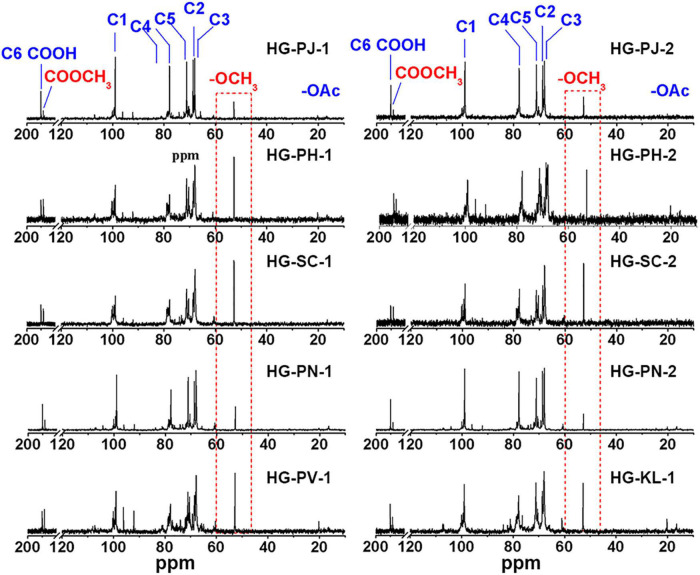
^13^C NMR spectrum of HG pectin.

^13^C-NMR was used to analyze the chemical structures of HG pectins (Figure 2). Signals at 99.36, 68.64, 67.97, 77.79, 71.08, and 175.04 ppm were assigned to the C-1, C-2, C-3, C-4, C-5, and C-6 atoms of →4)-α-D-GalA*p*-(1→, and the signal at 170.81 ppm was due to C-6 of →4)-α-D-MeGalA*p*-(1→, respectively. These chemical shifts suggested that HG is the dominant structure in these pectin fractions. The resonance at 52.74 ppm was attributed to the -OCH_3_ group, indicating that these HG pectins were methyl-esterified. The absence of a (or a very weak) signal at ∼19.16 ppm indicated that these HG pectins contained no (or only very small amounts of) acetyl groups. Aside from characteristic signals from the HG backbone, some weak signals at 98.91 and 16.44 ppm were identified as C-1 and C-6 of →2)-α-L-Rha*p*-(1→, respectively, and signals at 104.26 ppm and 106.92 ppm were from C-1 of β-D-Gal*p* and α-L-Ara*f*, respectively. These results indicated that few RG-I domains are also present in these fractions, consistent with monosaccharide composition. The major signals in ^13^C-NMR spectra of these HG pectins were similar, whereas the pectin fraction with a small RG-I content had a more complicated spectrum. In addition, signal intensities at 52.74 and 170.81 ppm (attributable to methyl and methyl ester groups, respectively) were different, suggesting that the degree of methyl-esterification (DM) may be different.

### FT-IR Analysis of the DM of HG Pectins

**FIGURE 3:**
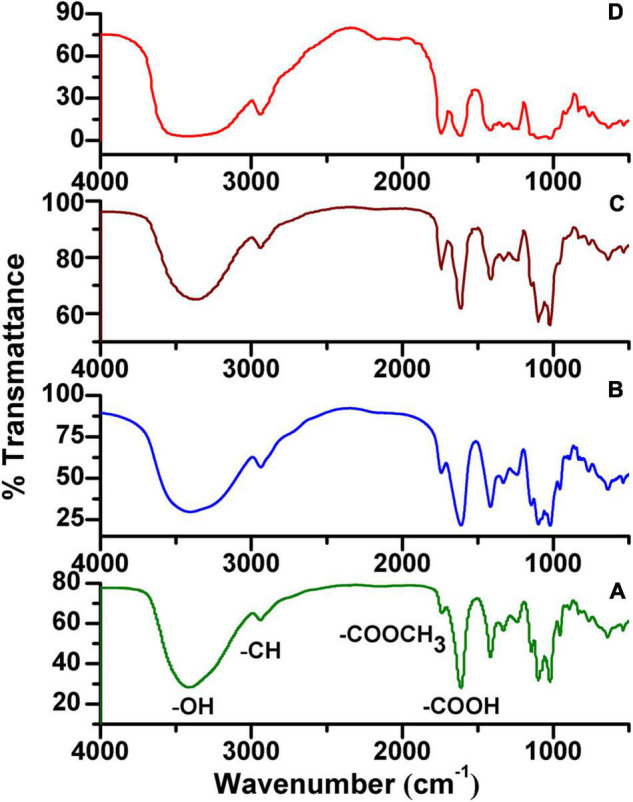
Fourier transform-infrared spectroscopy (FT-IR) spectra of HG pectins from plants. **(A)** HG-PN-2 (Class I); **(B)** HG-PJ-1 (Class II); **(C)** HG-CC-1 (Class III); and **(D)** HG-KL-1 (Class IV).

Based on our NMR analysis, these HG pectins were all methyl-esterified. To determine the DM of these HG pectins, FT-IR analysis was performed. The FT-IR spectra of 20 HG pectins (Figure 3 and Supplementary Figure 3) exhibited similar characteristic peaks from 400 to 4,000 cm^–1^, as well as absorption peaks at 1,740 and 1,630 cm^–1^ that were attributed to C = O vibrations of the methyl-esterified GalA and GalA in the acidic forms, respectively. However, the peak areas at 1,740 cm^–1^ and 1,630 cm^–1^ were distinct for some pectins. Based on these two peak areas, DM values were calculated as described in the “Methods” section. As shown in Table 2, the DM of these HG pectins was all less than 50%, suggesting that their HGs have relatively low methyl-esterification. According to DM values, these HG pectins were divided into four classes with DM values of (I) 0–10%, (II) 10–20%, (III) 20–30%, and (IV) more than 30%. We choose HG-PN-2, HG-PJ-1, HG-CC, and HG-KL-1 from each class of pectin as examples in the following methyl-esterification pattern analysis.

**TABLE 2:** The degree of methyl-esterification (DM) of 20 HG pectins.

Class	Fraction	DM (%)
I	HG-AC	5.0
	HG-PN-2	6.6
	HG-PJ-2	9.6
II	HG-PN-1	11.2
	HG-PJ-1	17.6
	HG-PV-2	19.8
III	HG-PH-2	21.8
	HG-AA-2	23.5
	HG-SF	23.7
	HG-PH-1	25.0
	HG-AA-1	25.2
	HG-PO-1	27.2
	HG-PO-2	27.7
	HG-CC	28.9
IV	HG-PV-1	30.3
	HG-KL-2	31.0
	HG-SC-2	33.5
	HG-SC-1	34.8
	HG-II	39.0
	HG-KL-1	40.2

### Quantitative and Methyl-Ester Distribution of Mono-/Oligo-Galacturonides

Due to the relatively large molecular weight distributions in HG pectins, specific sites of methyl-esterification are difficult to determine directly using chemical or spectroscopic approaches. Therefore, enzymatic hydrolysis was employed to degrade the HGs into mono-/oligo-galacturonides. By a quantitative analysis of nonesterified and esterified mono/oligo-galacturonides and by analyzing the structures of esterified oligogalacturonides, methyl-esterification patterns were estimated (38).

#### Endo-Polygalacturonase Hydrolysis of HG Pectins

Endo-PG was used to degrade HG pectins because they were only minimally esterified. Endo-PG [EC 3.2.1.15] is purified from *Aspergillus aculeatus* that can hydrolyze at least four continuous unesterified α-(1-4)-GalA linkages. This enzyme cannot hydrolyze glycosidic bonds between esterified α-(1-4)-GalA groups (39). The products of Endo-PG degradation were monitored using HPGPC (Supplementary Figure 4). Oligosaccharides with MW < 2 kDa were the major products. In addition, a small number of large and medium molecular weight fractions were also produced, which arise from RG-I and RG-II domains (34, 35, 40). Following degradation, hydrolysates were separated using Sephadex G-25 to remove polymers, thus yielding mono-/oligo-galacturonides (yield∼50–80%).

#### HILIC-FLR-MS Analysis of Mono-/Oligo-Galacturonides

**FIGURE 4:**
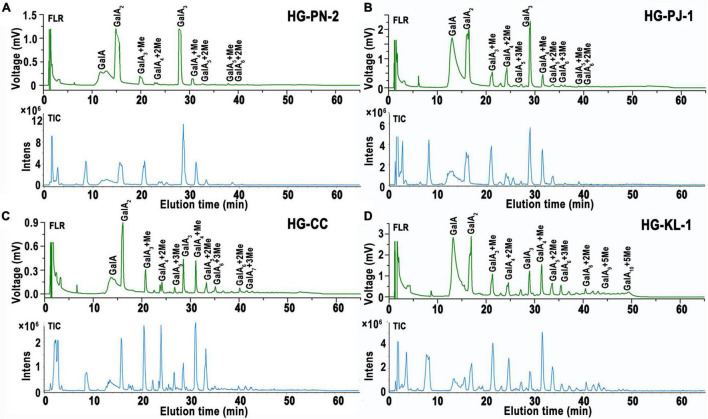
HILIC elution patterns of GalA-oligomers produced from HG pectins with different degrees of methyl-esterification (DM). **(A)** HG-PN-2 (Class I); **(B)** HG-PJ-1 (Class II); **(C)** HG-CC-1 (Class III); and **(D)** HG-KL-1 (Class IV).

In this study, 2-AB was used to label mono-/oligo-galacturonides that were produced by enzymatic hydrolysis of HG pectins. This labeling protocol stoichiometrically links one fluorescent probe per reducing end of an oligosaccharide, with labeling efficiency reaching greater than 85% (41). Fluorescently labeled mono-/oligo-galacturonides were completely separated by HILIC. In this study, four fractions were taken to exemplify our analytical approach. Figure 4 shows the total ion chromatogram (TIC) of the mono-/oligo-galacturonides by HILIC-FLR-MS analysis. The HILIC-MS elution pattern showed that the derivatized oligogalacturonides have masses lower than ∼1,200 exhibiting mostly [M-H]^–1^ ions, and oligomers greater than ∼1,200 were represented by both [M-H]^–1^ and [M-H]^–2^ ions. Nonesterified mono-/oligo-galacturonides were monomers (313), dimers (417), and trimers (647). Methyl-esterified oligogalacturonides with different DP values and different numbers of methyl-ester groups were observed, including DP 3–5 containing a single methyl-ester group, DP 4–6 containing two methyl-ester groups, DP 5–8 containing three methyl-ester groups, DP 7–9 containing four methyl-ester groups, DP 8–10 containing five methyl-ester groups, and DP 9–10 containing six methyl-ester groups.

#### Quantification of Mono-/Oligo-Galacturonides by HILIC-FLR Analysis

**FIGURE 5:**
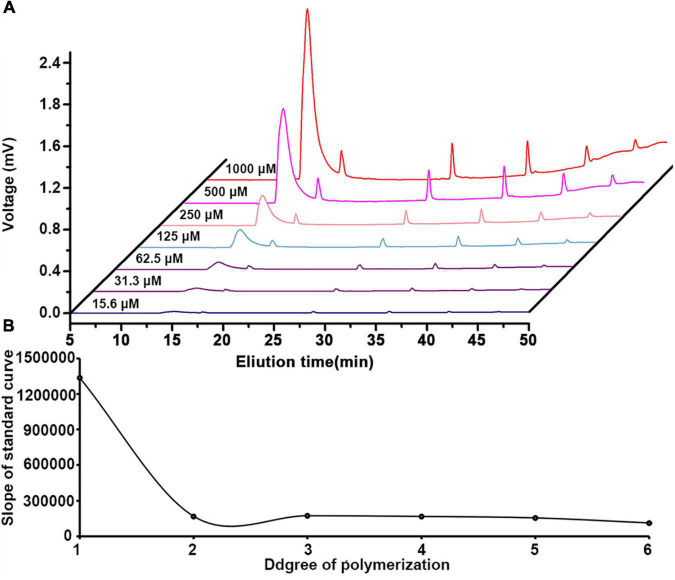
**(A)** HILIC-FLR elution curves of oligogalacturonide standards (DP1-6) at different concentrations. **(B)** Comparison of the slope of GalA standard curve.

To quantify the content of mono/oligo-galacturonides from different HG pectins, a set of standard mono-/oligo-galacturonides (DP1-6) at different concentrations were first analyzed by HILIC-FLR (Figure 5A). Calibration curves showed excellent linearity over the entire concentration range (15.6 to 1,000 μM) with *R*^2^ values falling between 0.9955 and 0.9996 (Supplementary Table 1). The limit of detection (LOD) and limit of quantitation (LOQ) ranges were within 0.1–0.3 and 0.4–1.2 μM, respectively (Supplementary Table 1). By comparing standard curves with different DPs, we found that the slope (*K*) of the GalA standard curve was the highest, whereas other oligomers (DP2-6) had slopes that tended to be relatively smooth (Figure 5B). Therefore, we used the DP6 standard curve to calculate the content of oligomers with DP > 6. As methyl-esterified oligogalacturonide standards were not available, the content of methyl-esterified oligogalacturonide was estimated using a standard curve of nonesterified oligogalacturonides with the same DP. The molar percentage of mono-/oligo-galacturonides from different HG pectins in HILIC-FLR was calculated using standard curves, and the results are shown in Supplementary Table 2. Among these mono-/oligo-galacturonides, nonesterified monomers, dimers, and trimers had the largest amounts of total mono-/oligo-galacturonides. The variety of oligogalacturonides from HG pectin with higher DM values was more abundant than those with lower DM values, and the content of oligogalacturonides decreased gradually along with the increase in DP.

#### Methyl-Ester Group Distribution in Oligogalacturonides

To elucidate sites of methyl-esterification in HG pectin oligosaccharides, we used MS^2^ fragmentation analysis. The reducing end of these oligosaccharides was labeled with the fluorescent probe 2-AB. Using the negative mode MS detection, we found that the cleavage of Z and Y ions increased by 102 over the *m*/*z* value, whereas C and B ions remained unchanged. Fragmentation patterns were annotated according to Domon and Costello (42). We used HILIC-MS^2^ to analyze 18 oligogalacturonides with different numbers of methyl-ester groups, six of which are analyzed in detail and shown below.

**FIGURE 6:**
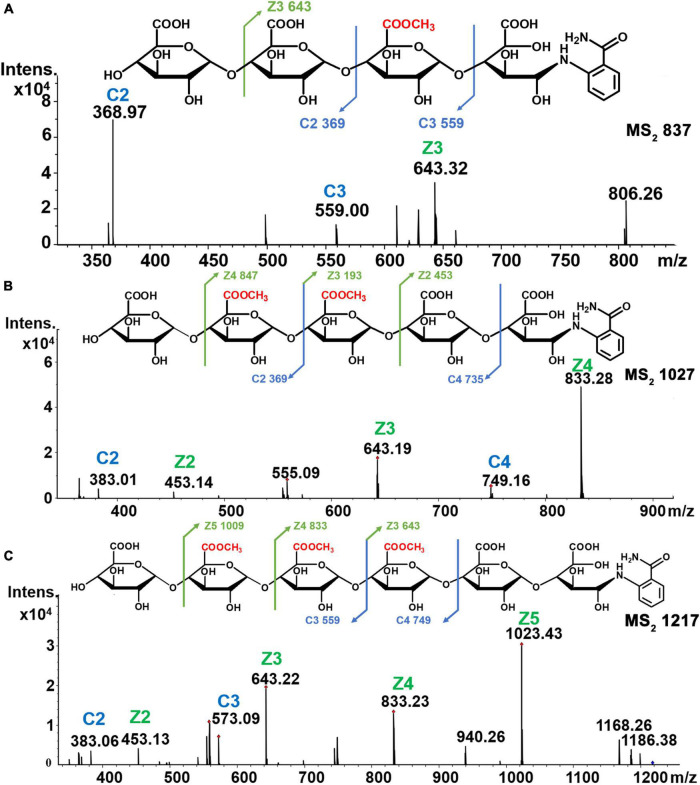
Chemical structures and MS_2_ spectra of oligogalacturonides of different methyl groups. **(A)** Mono-esterified of DP4. **(B)** Di-esterified of DP5. **(C)** Tri-esterified of DP6.

Mass spectrometry results demonstrated that the [M-H]^–1^ ion at *m*/*z* 819 corresponded to the mono methyl-esterified tetramer of galacturonic acid, and fragment peaks at *m*/*z* 369 [C2]^–^, *m*/*z* 559 [C3+Me]^–^, and *m*/*z* 643 [Z3+Me]^–^ in MS^2^ analysis confirmed the structure as GalA-GalA-GalA_Me_-GalA-2AB (Figure 6A). For di-methyl-esterified pentamers of galacturonic acid (*m*/*z* 1,027 [M-H]^–^ ion), fragment peaks at *m*/*z* 383 [C2+Me]^–^ and *m*/*z* 749 [C4+2Me]^–^ and their complementary *m*/*z* 453 [Z2]^–^, *m*/*z* 643 [Z3+Me]^–^, and *m*/*z* 833 [Z4+3Me]^–^ in MS^2^ spectra were observed. These data indicated that the oligogalacturonide sequence was GalA-GalA_Me_-GalA_Me_-GalA-GalA-2AB (Figure 6B). The [M-H]^–^ ion at *m*/*z* 1,217 corresponded to a tri-methyl-esterified hexamer of galacturonic acid. Fragment peaks at *m*/*z* 383 [C2+Me]^–^, *m*/*z* 573 [C3+2Me]^–^ and their complementary *m*/*z* 453 [Z2]^–^, *m*/*z* 643 [Z3+Me]^–^, *m*/*z* 833 [Z4+2Me]^–^, and *m*/*z* 1,023 [Z5+3Me]^–^ in MS^2^ spectra were found, suggesting the oligogalacturonide sequence of GalA-GalA_Me_-GalA_Me_-GalA_Me_-GalA-GalA-2AB (Figure 6C).

**FIGURE 7:**
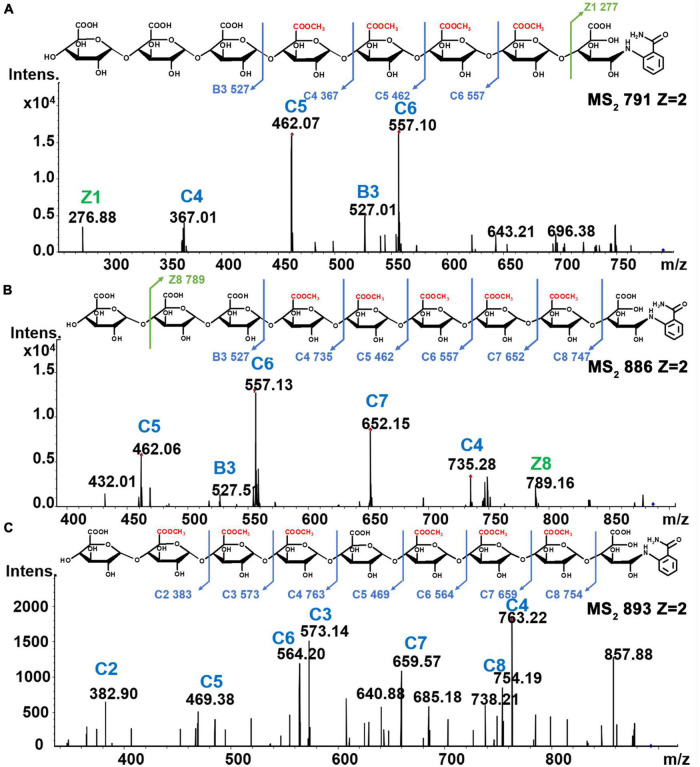
**(A)** Tetra-esterified of DP8 **(B)** penta-esterified of DP9 and **(C)** hex-esterified of DP9.

Oligomers having molecular weights greater than ∼1,200 Da showed mostly di-deprotonated [M-2H]^2–^ ions in MS^2^ spectra, such as the single-charged *m*/*z* 1,583 [M-H]^–^ ion and the di-deprotonated species at *m*/*z* 791 [M-H]^2–^ ion associated with the tetra-esterified octamer of galacturonic acid. Furthermore, the presence of fragment ions at *m*/*z* 527 [C3]^–^, *m*/*z* 367 [C4+Me]^2–^, *m*/*z* 462 [C5+2Me]^2–^, *m*/*z* 557 [C6+3Me]^2–^, and *m*/*z* 277 [Z1]^–^ in MS^2^ spectrum confirmed the sequence of the heptamer as GalA-GalA-GalA-GalA_Me_-GalA_Me_-GalA_Me_-GalA_Me_-GalA-2AB (Figure 7A). The penta-methyl-esterified nonamer (*m*/*z* 886 [M-H]^2–^) showed peaks at *m*/*z* 527 [B3]^–^, *m*/*z* 735 [C4+Me]^–^, *m*/*z* 462 [C5+2Me]^2–^, *m*/*z* 557 [C6+3Me]^2–^, *m*/*z* 652 [C7+4Me]^2–^, and *m*/*z* 747 [C8+5Me]^2–^ in MS^2^ spectra, and the structure was determined to be GalA-GalA-GalA-GalA_Me_-GalA_Me_-GalA_Me_-GalA_Me_-GalA_Me_-GalA-2AB (Figure 7B). The hexa-methyl-esterified nonamer (*m*/*z* 893 [M-H]^2–^) with peaks at *m*/*z* 383 [C2+Me]^–^, *m*/*z* 573 [C3+2Me]^–^, *m*/*z* 763 [C4+3Me]^2–^, *m*/*z* 469 [C5+4Me]^2–^, *m*/*z* 564 [C6+4Me]^2–^, *m*/*z* 659 [C7+5Me]^2–^, and *m*/*z* 754 [C8+6Me]^2–^ in MS^2^ spectra, having the structure of GalA-GalA_Me_-GalA_Me_-GalA_Me_-GalA-GalA_Me_-GalA_Me_-GalA_Me_-GalA-2AB (Figure 7C). The structures of another 12 different methyl-esterified oligogalacturonides are shown in Supplementary Figure 4.

Based on these analyses, we could see that the most continuously distributed methyl-ester group was five in these oligomers. With the increase in DP of the oligogalacturonides, 1-2 GalA residue gaps also existed between methyl ester groups. Oligomers carrying a maximum of 3 consecutive methyl-ester groups have been reported (19, 43). Pectin lyase (EC 4.2.2.10), which cleaves between methyl-esterified GalA residues in HG regions by β-elimination, was used (together with Endo-PG in some studies) to analyze high DM pectins (19, 20) that may result in a decrease of consecutive methyl-ester groups in GalA-oligomers. Due to the low DM values of these 20 HG pectins, pectin lyase was not used in our study. During our analysis of the methyl-ester group sites in these oligogalacturonides, there may be characteristic mass spectrum peaks of the corresponding isomers; however, their fine structures could not be resolved due to having only small amounts of material.

### Methyl-Esterification Pattern of 20 HG Pectins

**FIGURE 8:**
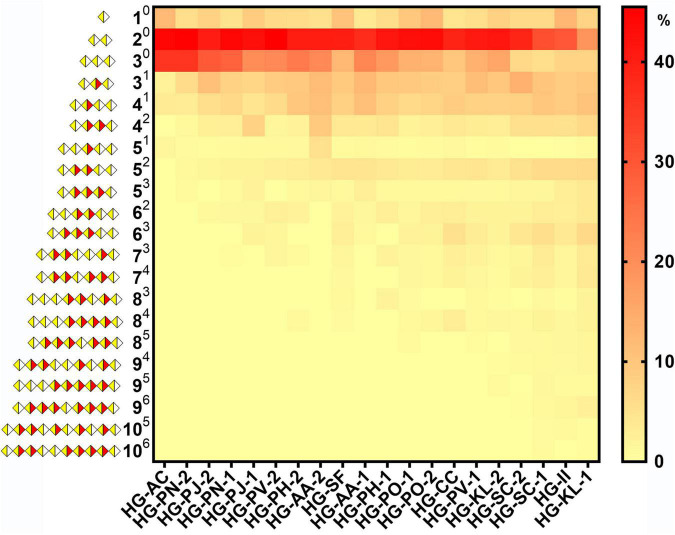
The heatmap of species and quantification of HG pectin oligosaccharides in different plants (A*^B^* A is the degree of galacturonic acid; B is the number of methyl groups; 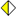 GalA; 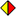 methylesterified GalA).

Quantification and distribution of methyl-ester groups in pectin-derived HG oligosaccharides with different DM values were systematically investigated (Figure 8). We found that large amounts of nonesterified monomers, dimers, and trimers were released on Endo-PG degradation. Monomers and dimers were only present as nonesterified oligomers, consistent with previous studies (38, 44). For the total amount of nonesterified oligomer, the higher the DM of the pectin, the smaller the percentage of trimmer. This suggested a gradual decrease in the average size of nonesterified GalA fragments in HG pectins with DM values increasing. In addition, the higher DM of the pectin, the larger the amount of methyl-esterified oligomers. We noticed that mono- and di-esterified oligomers had a larger percentage in total methyl-esterified oligomers, and the amounts and types of methyl-esterified oligomers were quite abundant in higher DM pectins, indicating a relatively random pattern of methyl-ester distribution in these 20 HG pectins (19). Although the plant PMEs were reported as a kind of pectin methylesterase [leading to the appearance of de-esterified stretches or blocks (20, 45)], we assumed that the de-esterification of pectins in plants is a more complex process than using various PMEs (46). Furthermore, although some of these twenty HG pectins were purified from plants and used in medicines and foods, others were derived from plants and used only for drugs. We noticed that there was no difference between their structural features, including methyl-esterification distribution patterns. This suggests that toxic side effects from medicinal plant products may not be caused by their HG pectins and that these HG pectins may be safely used.

**FIGURE 9:**
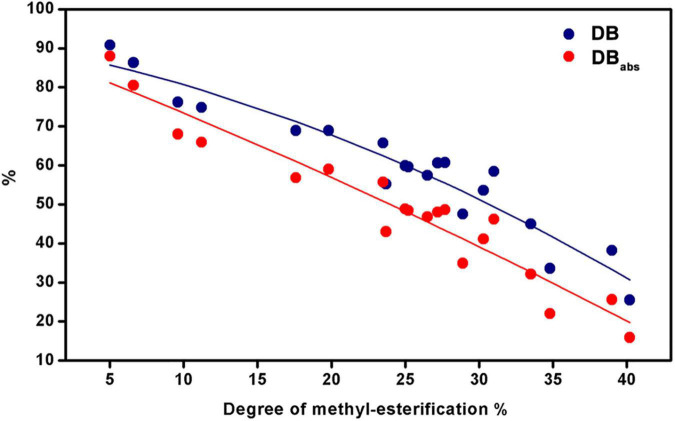
DB and DB_abs_ as a function of the degree of methylesterification for the 20 HG pectins.

Furthermore, the DB and DB_abs_ values of 20 HG pectins were calculated to characterize their methyl-esterification patterns (Supplementary Table 3). In this study, we found a gradual decrease in DB or DB_abs_ values with increasing DM values (Figure 9). These DB and DB_abs_ values indicate a random or blockwise pattern of methyl-esterification. Pectins with similar DM values, but with different DB or DB_abs_ values, may have a nonidentical pattern of methyl-esterification (38, 47). Among the pectins with smaller DM values, DB or DB_abs_ values were relatively close to each other, suggesting similar degrees of methyl-esterification (Figure 9). It has been reported that DM and DB values of HG pectins are both related to function. Low DM pectins (DM≈20) and intermediate DM pectins (DM≈50) with high DB values (DB60) can strongly inhibit TLR2-1. DM50 pectins with low DB values (DB33) and those with high DM values (DM≈80) did not inhibit TLR2-1 (16, 17). Moreover, DM20 pectins with high DB values (DB94), as well as DM50 pectins, showed a capacity to regulate T cell-based immunity, whereas DM20 pectins with low DB values (DB86) did not (18). Therefore, HG pectins with various DM and DB values may well possess diverse functions, something that needs to be further explored in the future.

**FIGURE 10:**
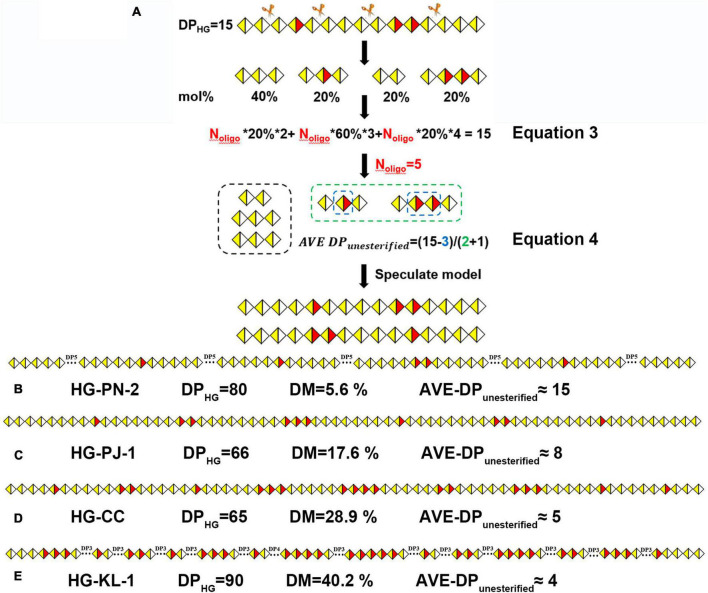
**(A)** The calculation process of the number of oligogalacturonides (*N*_*oligo*_); methyl-esterification distribution models of HG pectins with different DM: **(B)** HG-PN-2(Class I); **(C)** HG-PJ-1 (Class II); **(D)** HG-CC (Class III); **(E)** HG-KL-1 (Class IV).

According to our results, the average size of nonesterified GalA regions in these pectins could not be obtained using DB and DB_abs_ values because their DP values and degree and position of methyl-esterification were not considered. To better illustrate the methyl-esterification pattern in HG pectins, we have established a new approach. By using a model HG pectin with a degree of polymerization (DP_HG_) of 15 as an example (Figure 10A), four types of oligogalacturonides, namely GalA-GalA (20%), GalA-GalA-GalA (40%), GalA-GalA_Me_-GalA (20%), and GalA-GalA_Me_-GalA_Me_-GalA (20%), were obtained on Endo-PG digestion. The total amount of enzymatically hydrolyzed HG oligosaccharides (*N*_oligo_) was calculated using DP_HG_, the molar percentage of each oligogalacturonide, and its corresponding DP value (equation 3). Each amount of resulting oligogalacturonide (equal to *N*_oligo_ multiplied by the molar percentage) was calculated as 1, 2, 1, and 1, respectively. We defined GalA_Me_ in each oligogalacturonide as a “block,” and unesterified GalA was equally distributed between “blocks.” The average size of unesterified GalA (AVE DP_unesterified_) imbedded in the “blocks” was calculated from the difference in DP_HG_ and GalA_Me_ values divided by the “block” amount (*N*_block_) plus one (equation 4). The AVE DP_*unesterified*_ was four in this distribution model, allowing two models to be inferred (Figure 10A).


(3) $$N_{o\,l\,i\,g\,o}=\frac{D\,P_{H\,G}}{\sum_{n=1-10}\left[O\,l\,i\,g\,o\,G\,a\,l\,A_{n}\%\right]\times n}$$

(4) $$A\,V\,E\,D\,P_{u\,n\,e\,s\,t\,e\,r\,i\,f\,i\,e\,d}\approx \left(D\,P_{H\,G}-G\,a\,l\,A_{M\,e}\right)/\left(N_{b\,l\,o\,c\,k}+1\right)$$

Using this approach, we analyzed the methyl-ester distribution pattern for Class IV (HG-KL-1). HPSEC-RI-MALLS showed that the MW of HG-KL-1 is 30 kDa, with the DP_HG_ value being calculated (MW × GalA % of monosaccharide)/176 as ∼90. Different mono-/oligo-galacturonides were quantified using HILIC-FLR (Supplementary Table 2), and approximately 20 oligogalacturonide fragments were calculated according to equation 3 (*N*_oligo_ = 20). The product of *N*_oligo_ × molar percentage yielded the number of GalA, GalA-GalA, and GalA-GalA-GalA blocks in these oligosaccharides, which were found to be 1, 4, and 1, respectively. Similarly, the “blocks” containing 1–5 esterified groups were calculated to be 5, 3, 3, 2, and 1, respectively, reaching *N*_block_ = 14 along with GalA_Me_ = 33. In this regard, the AVE DP_unesterified_ was estimated to be 4. The proposed model for HG-KL-1 was established using these results (Figure 10E), along with the models of the other three classes of HG pectins (Figures 10B–D).

Among our 20 HG pectins, the ratio of *N*_block_/*N*_oligo_ was larger in higher DM pectins. The AVE DP_*unesterified*_ of these four classes of HG pectins was calculated as a range of 18–15, 12–8, 8–5, and 6–4, respectively, suggesting that the average size of nonesterified GalA regions in these pectins was decreased on increasing DM values (Figure 10 and Supplementary Table 3). We also calculated DM values based on these models, with results being consistent with FT-IR data. In general, DB values could not provide information as to whether the pectin may contain one large block or several smaller blocks of nonesterified GalA residues. When two pectins have similar DM and DB values, but different functions, it becomes difficult to use DB values to explain the relationship between methyl-ester distribution and function. In contrast, the parameter AVE DP_*unesterified*_ could be introduced to analyze methyl-ester distribution and function of the pectin.

Nevertheless, some limitations to our approach remain. First, our analytical strategy is only suitable for use with low methyl-esterified HG pectins. For the methyl-ester group distribution analysis with high DM pectins, PL should be used with Endo-PG completely degrading the pectins (48). Furthermore, we assumed that blocks are evenly separated in our models, whereas the actual distribution of esterified-GalA residues is likely to be more complex, and the sequence of different block lengths remains uncertain. In this study, we only showed one type of random distribution model. Nevertheless, we identified a class of oligosaccharides with discontinuous methyl-ester groups using HILIC-FLR-MS^n^ analysis, but these did not show up in our methyl-esterification distribution models due to their low content.

## Conclusion

In this study, 20 HG pectins were prepared from twelve plants. The DM of these HG pectins ranged from 5 to 40%. Enzymatic fingerprinting of HG pectins with different DM values was performed by enzymatic hydrolysis, fluorescence labeling of mono/oligo-galacturonides, and HILIC-FLR-MS^n^ analysis. According to the quantitative and methyl-esterification distribution analysis of oligogalacturonides, we inferred the random methyl-esterification pattern in these HG pectins and proposed possible models for them. Our study reveals the characteristics of methyl-esterification patterns in natural HG pectins from plants and provides the basis for developing their structure-function relationships. In turn, our findings can be applied to the use of HG pectins in healthy foods.

## Data Availability Statement

The original contributions presented in the study are included in the article/Supplementary Material, further inquiries can be directed to the corresponding author/s.

## Author Contributions

YY: investigation and writing—original draft. LC, XL, and YW: investigation. CS: formal analysis. UP: modify the manuscript. KM, LS, and YZ: writing—review and editing. All authors contributed to the article and approved the submitted version.

## Conflict of Interest

The authors declare that the research was conducted in the absence of any commercial or financial relationships that could be construed as a potential conflict of interest.

## Publisher’s Note

All claims expressed in this article are solely those of the authors and do not necessarily represent those of their affiliated organizations, or those of the publisher, the editors and the reviewers. Any product that may be evaluated in this article, or claim that may be made by its manufacturer, is not guaranteed or endorsed by the publisher.
