# Supplementary material for: Determining Methyl-Esterification Patterns in Plant-Derived Homogalacturonan Pectins
Source: Front Nutr. 2022 Jul 1;9:925050. doi: 10.3389/fnut.2022.925050 (PMC9330511; doi:10.3389/fnut.2022.925050)
Supplement: Supplementary file 1 [file Data_Sheet_1.docx]

Supplementary Material

**1 Supplementary Method**

**S.1 UV-vis spectroscopy**

A polysaccharide sample (2 mg) was dissolved in distilled water (4 mL). UV–vis absorption spectra (190 nm to 800 nm) were recorded using a Shimadzu UV-2700 spectrophotometer.

**S.2 Determination of molecular weight distribution**

Molecular weight distribution curves were determined by using high performance gel-permeation chromatography (HPGPC) with a TSK-gel G-3000 PWXL column (7.8×300mm, TOSOH, Japan) coupled to a Shimadzu high performance liquid chromatography (HPLC) system (Tokyo, Japan) (1).

**S.3 Monosaccharide composition analysis**

Monosaccharide composition was determined by using high performance liquid chromatography (HPLC) as previously described (1). Briefly, polysaccharide samples (1 mg) were hydrolyzed by using hydrochloric acid/methanol prior to treatment with trifluoroacetic acid. Hydrolysates were derived using 1-phenyl-3-methyl-5-pyrazo-lone (PMP) and detected by HPLC (UV–VIS detector) equipped with C18 column (DIKMA Inertsil ODS-3 column 4.6 mm × 150 mm).

**S.4 ^13^C NMR spectra analysis**

A 20 mg sample was deuterium-exchanged and dissolved in 0.5 mL of D_2_O (99.8 %). ^13^C NMR spectra were measured at 20 ℃ on a Bruker 600 MHz NMR spectrometer (Karlsruhe, Germany) operating at a frequency of 150 MHz. Data were analyzed using standard Bruker software.

**S.5 HILIC-FLR/ESI-MS^n^ elution procedures and detection methods**

Diluted samples were filtered using a 0.45 μm Millipore membrane and injected into the HILIC-FLR/ESI-MSn. Elution was performed at a flow rate of 0.3 mL/min at 35 ℃, with a 5 μL injection volume. The fluorescence detector (FLD) with λ_ex_ of 330 nm and λ_em_ of 420 nm, was connected online prior to mass spectrometry (MS) analysis. ESI-MS detection was performed in the negative mode with a capillary voltage of 4500 V, capillary temperature of 200 °C and dry gas flow rate of 6 L/min. Mass spectra were acquired over the scan range of m/z 50 to 2000. Data were processed using Trap-control software.

The composition of the three mobile phases was (A) ultrapure water, (B) acetonitrile and (C) 200 mM ammonium formate with 50 mM formic acid under pH 3.0. The following elution profiles were used: 0-5 min, isocratic, 5% A, 90% B; 5-50 min, linear from 90 % to 60 %; B: the mobile phase of C was 5 % from beginning to end, followed by column re-equilibration: 50.1-55 min, linear from 60 % to 55 % A; 55-65 min, isocratic 5 % A, 90 % B and C 5 %.

**S.6 Quantification of oligogalacturonides**

The degree of polymerization of galacturonic acid oligomers (DP1-6) were determined by dissolution in a 50 % acetonitrile aqueous solution as the standard stock solution at the concentration of 10 mM. Serial dilutions were prepared to obtain a seven-point calibration curve at concentrations of: 1000 μM, 500 μM, 250 μM, 125 μΜ, 62.5 μM, 31.3μM, and 15.6 μM. Derivatization and HILIC-FLR methods used were the same as that of HG pectins from plants. Each group of standards was injected in triplicate (n=3), and then calibration curves were constructed. We continued to dilute the standards that were injected onto the HILIC-FLR instrument to determine the detection limit (LOD) and quantification limit (LOQ). Detection was limited to a signal-to-noise ratio of 3 (S/N = 3), and the quantification limit to a signal-to-noise ratio of 10 (S/N=10) (1).

**REFERENCES**

1. Zhang X, Yu L, Bi H, Li X, Ni W, Han H et al. Total fractionation and characterization of the water-soluble polysaccharides isolated from Panax ginseng C. A. Meyer. *Carbohydr. Polym.* (2009). 77:544-552. doi: 10.1016/j.carbpol.2009.01.034
2. Li Y, Liang J, Gao JN, Shen Y, Kuang HX, Xia YG. A novel LC-MS/MS method for complete composition analysis of polysaccharides by aldononitrile acetate and multiple reaction monitoring. *Carbohydr. Polym.* (2021), 272:118478. doi: 10.1016/j.carbpol.2021.118478

**2 Supplementary Figures**

**Supplementary Figure 1**


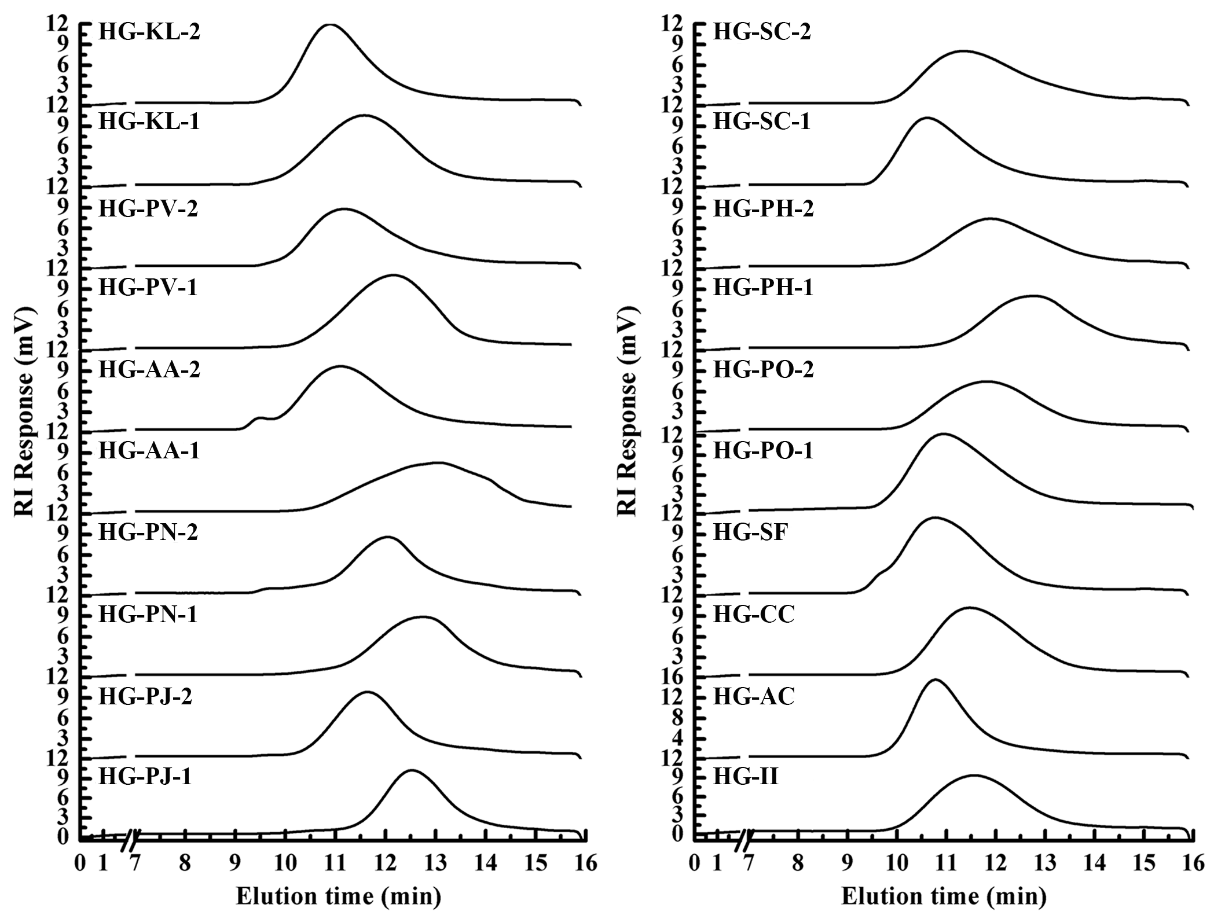


**Figure S1** HPSEC-RI elution curve of twenty kinds of HG pectins

**Supplementary Figure 2**

**
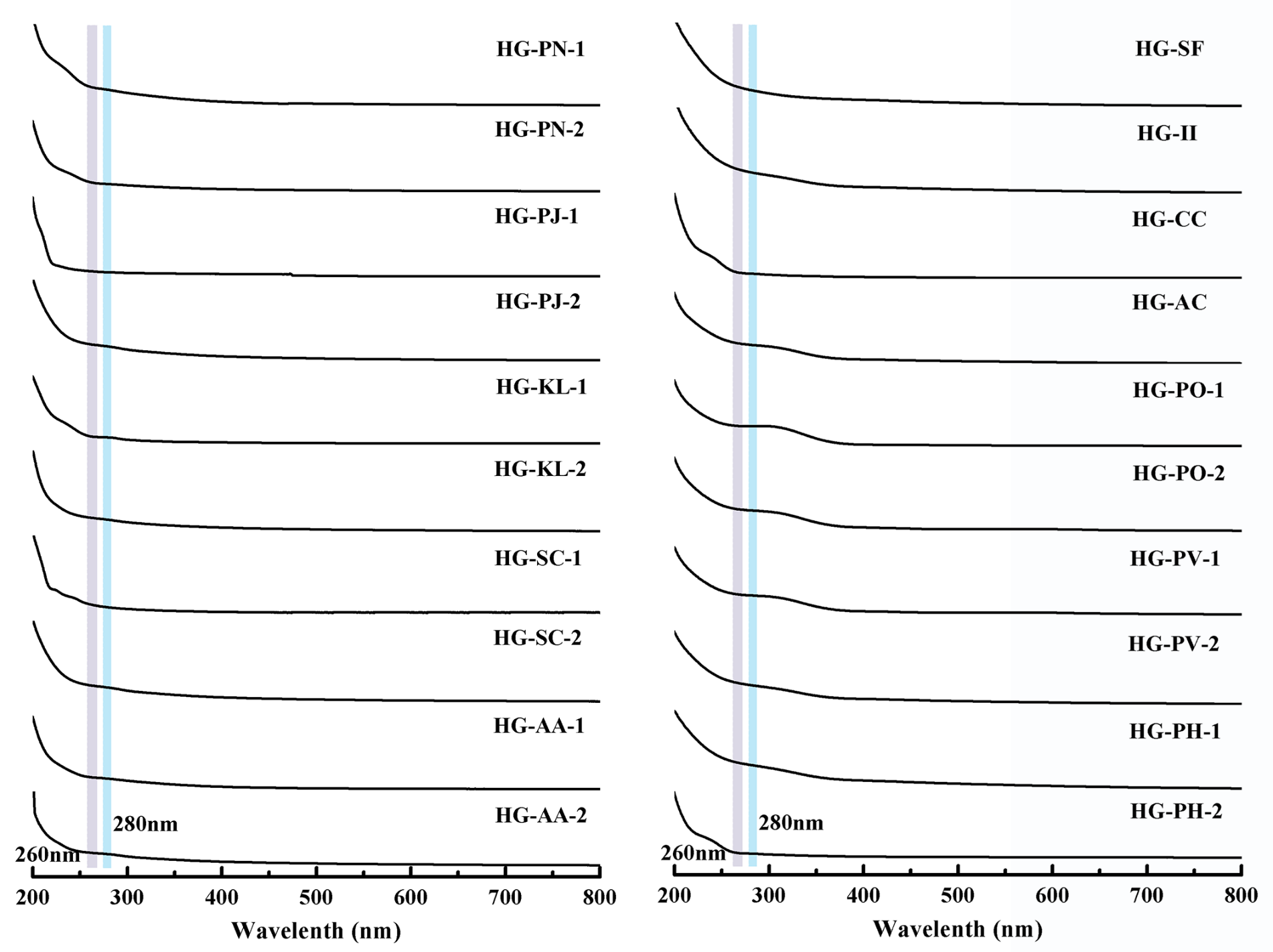
**

**Figure S2** UV-vis spectra of twenty kinds of HG pectins

**Supplementary Figure 3**


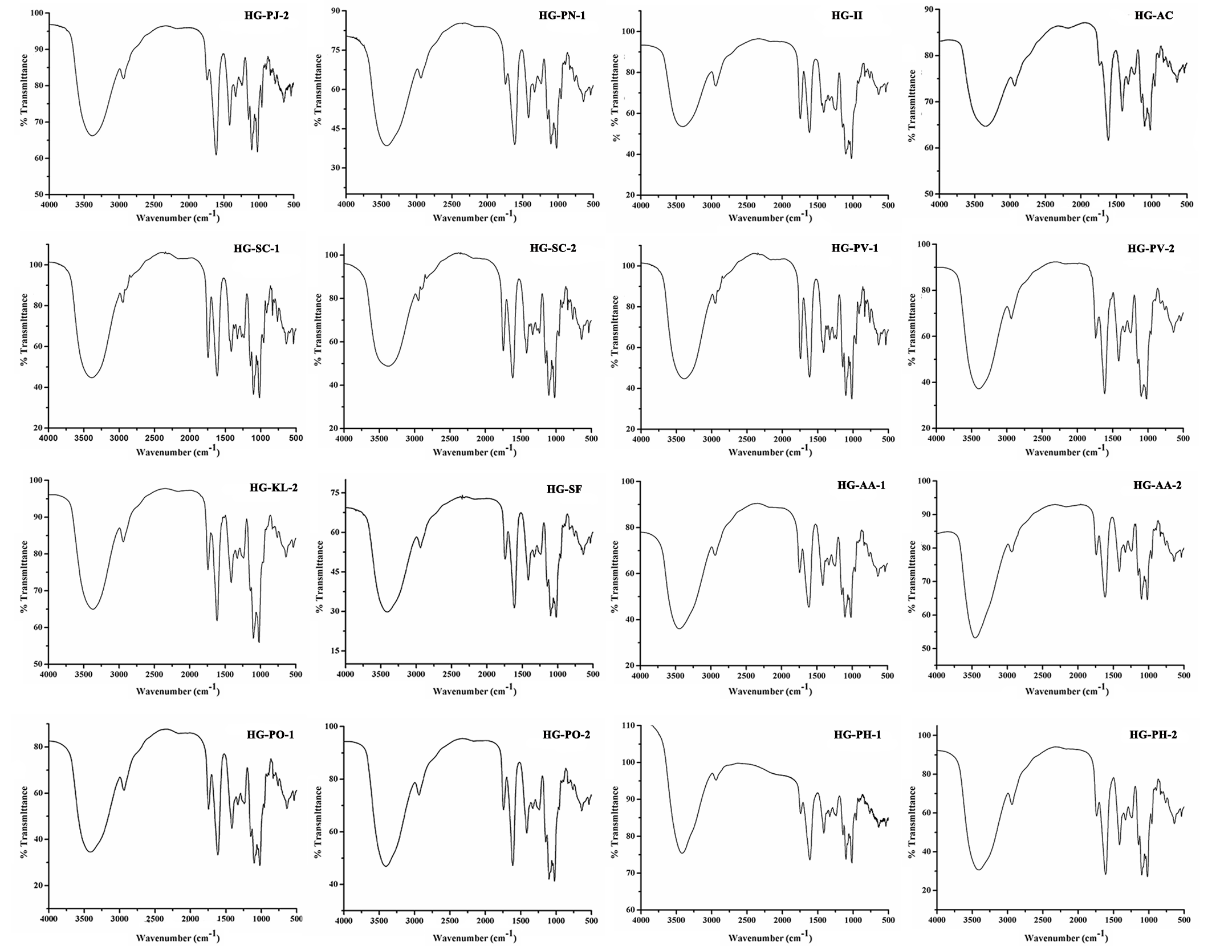


**Figure S3** FT-IR spectrum of other16 kinds of HG pectin.

**Supplementary Figure 4**


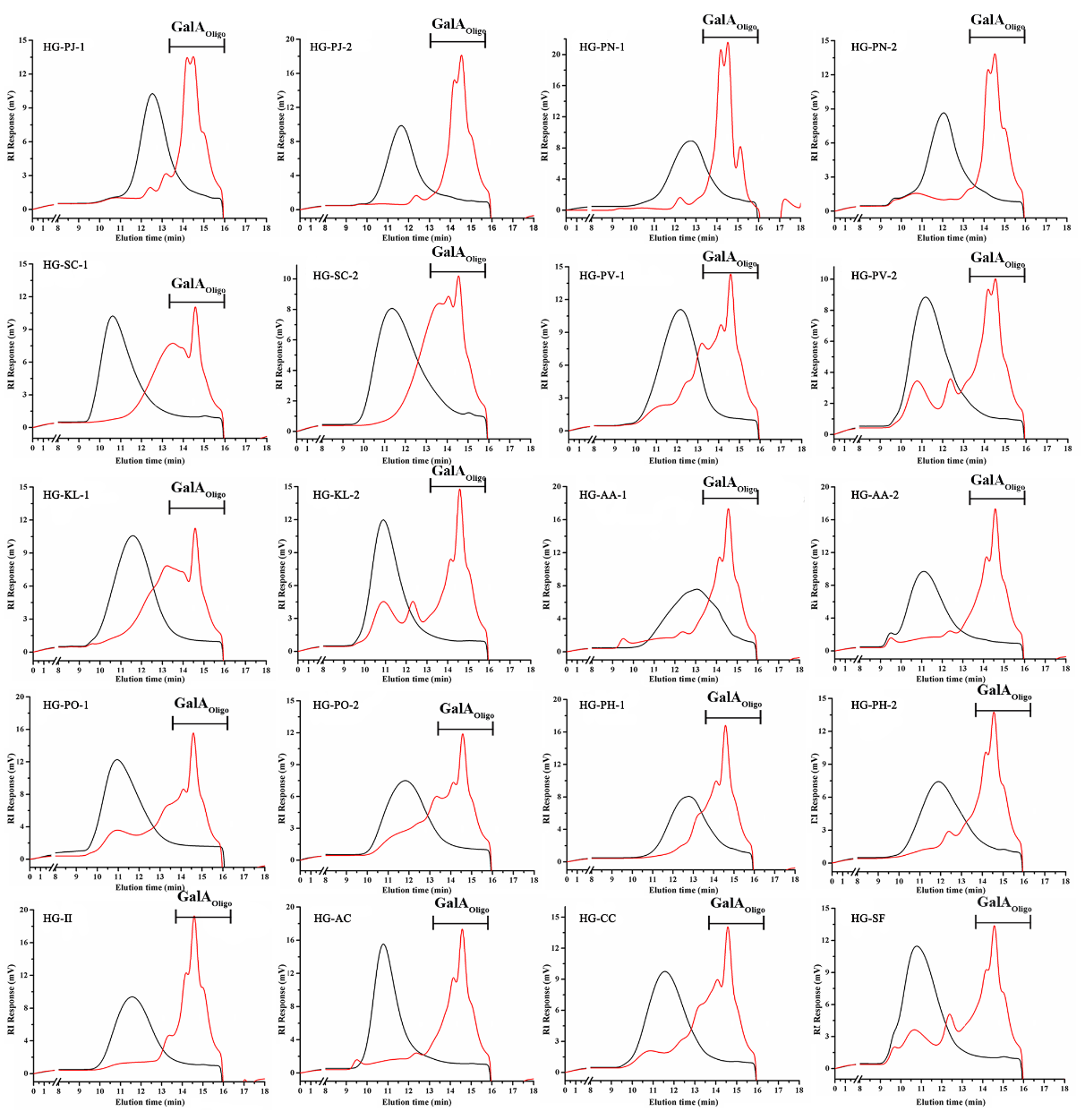


**Figure S4** Molecular weight distribution curves of 20 type of HG pectin after Endo-PG enzymatic hydrolysis (＿Before digestion；＿After digestion).

**Supplementary Figure 5**

**
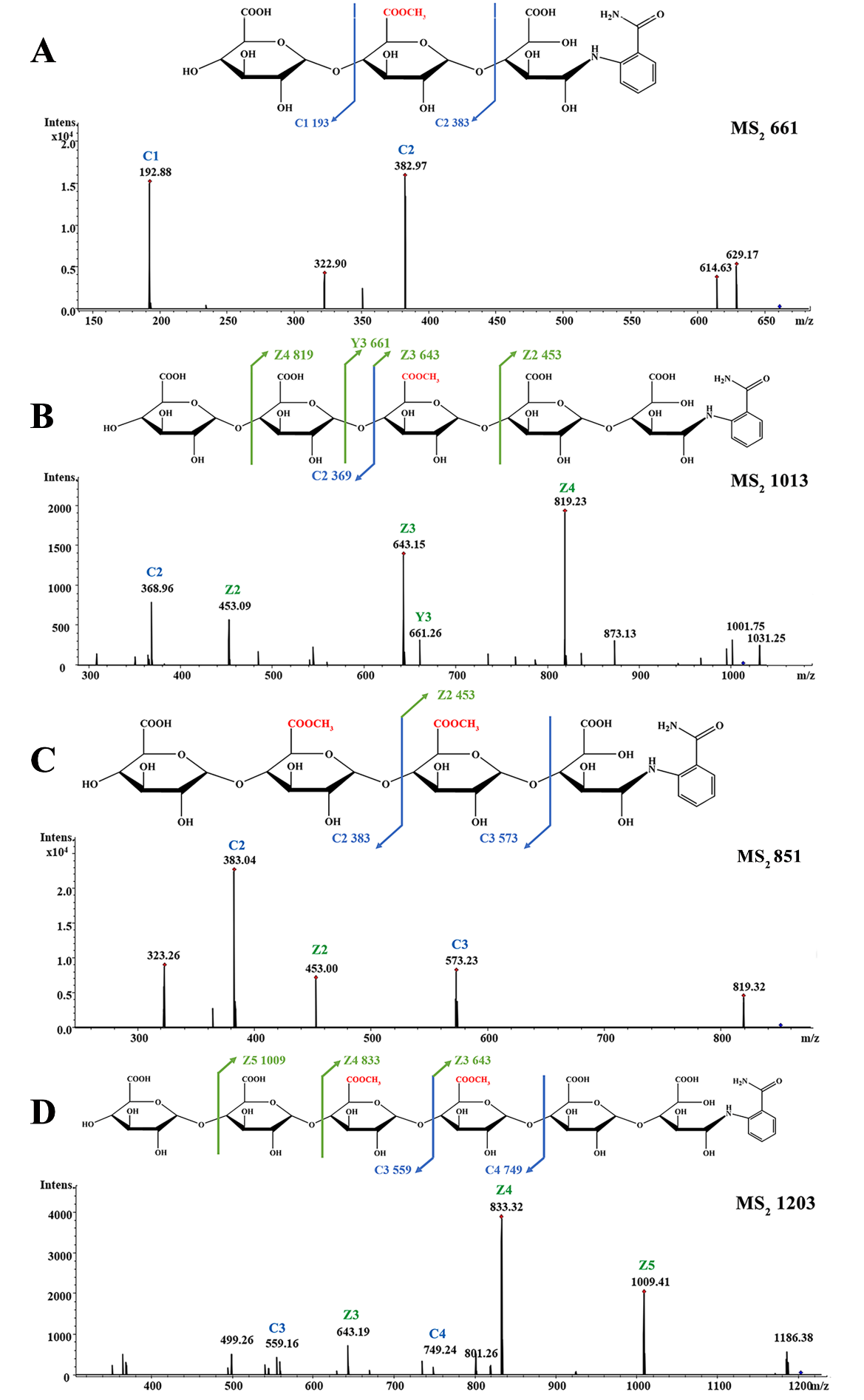
**

**Figure S****5** Chemical structures and MS_2_ spectra of other twenty type of oligogalacturonides. (A) mono-esterified of DP3 (B) mono-esterified of DP5 (C) di-esterified of DP4. (D) di-esterified of DP6

**
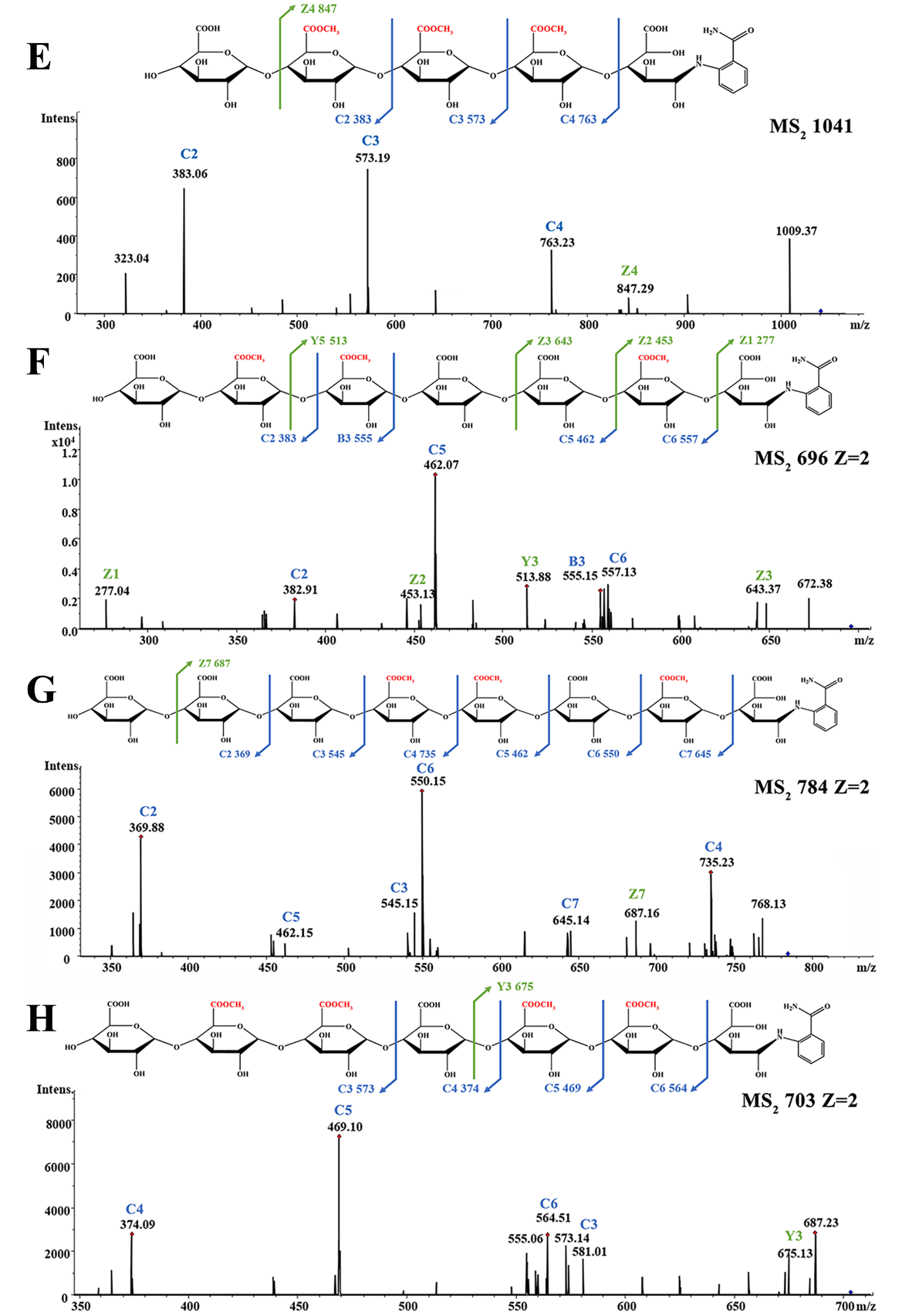
**

**Figure S5** (Continued) (E) tri-esterified of DP5 (F) tri-esterified of DP7 (G)tri-esterified of DP8 (H) tetra-esterified of DP7


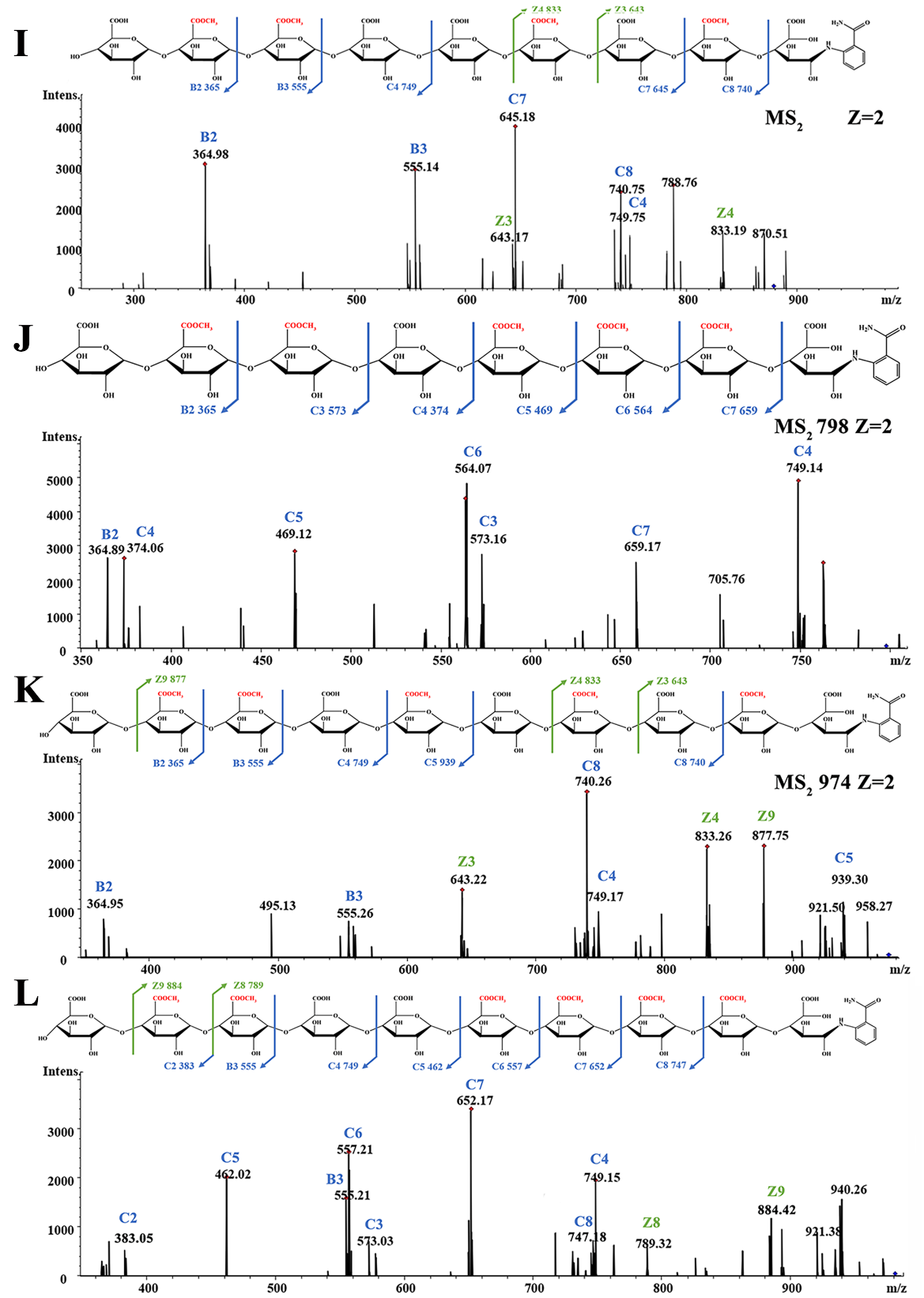


**Figure S5** (Continued) (I) tetra-esterified of DP9 (J) penta-esterified of DP8 (K) penta-esterified of DP10 (L) heax-esterified of DP6

**3 Supplementary Table**

**Supplementary Table 1** The standard curve and limits of detection and quantitation for DP 1-6 oligogalacturonide standards

| Oligomers | Linearity | | | LOD  (μM) | LOQ  (μM) |
| --- | --- | --- | --- | --- | --- |
|  | Calibration curve | Range (μM) | R^2^ |  |  |
| DP1 | Peak area=1335968C | 15.625-1000 | 0.9955 | 0.1 | 0.4 |
| DP2 | Peak area=167526C | 15.625-1000 | 0.9995 | 0.2 | 0.8 |
| DP3 | Peak area = 171905C | 15.625-1000 | 0.9996 | 0.2 | 0.8 |
| DP4 | Peak area = 165995C | 15.625-1000 | 0.9995 | 0.2 | 0.8 |
| DP5 | Peak area = 153899C | 15.625-1000 | 0.9983 | 0.1 | 0.4 |
| DP6 | Peak area=101318C | 15.625-1000 | 0.9989 | 0.3 | 1.2 |

**Supplementary Table 2** The quantify result of oligosaccharide fragments of HG pectins with different DM by fluorescence labeling

| DP | m/z | Structure | Molar percentage (%) | | | |
| --- | --- | --- | --- | --- | --- | --- |
|  |  |  | HG-PN-2 | HG-PJ-1 | HG-CC | HG-KL-1 |
| DP1 | 313 | GalA~☆^b^ | 5.7 | 8.9 | 4.6 | 7.6 |
| DP2 | 471 | 2GalA~☆ | 45.3 | 42.7 | 39.3 | 18.9 |
| DP3 | 647 | 3GalA~☆ | 35.9 | 22.7 | 10.1 | 7.5 |
|  | 661 | 3GalA+Me~☆ | 6.1 | 7.3 | 8.4 | 9.8 |
| DP4 | 837 | 4GalA+Me~☆ | 3.3 | 4.6 | 8.6 | 10.4 |
|  | 851 | 4GalA+2Me~☆ | 1.2 | 5.8 | 3.8 | 6.7 |
| DP5 | 1013 | 5GalA+Me~☆ | 0.8 | 0.9 | 1.1 | 0.6 |
|  | 1027 | 5GalA+2Me~☆ | 1.2 | 2.1 | 4.4 | 6.6 |
|  | 1041 | 5GalA+3Me~☆ | 0.6 | 1.8 | 1.2 | 3.6 |
| DP6 | 601 Z=2^a^ | 6GalA+2Me~☆ |  | 0.9 | 3.0 | 3.7 |
|  | 608 Z=2^a^ | 6GalA+3Me~☆ |  | 2.3 | 5.2 | 6.7 |
| DP7 | 696 Z=2^a^ | 7GalA+3Me~☆ |  |  | 2.4 | 3.5 |
|  | 703 Z=2^a^ | 7GalA+4Me~☆ |  |  | 2.0 | 3.8 |
| DP8 | 784 Z=2^a^ | 8GalA+3Me~☆ |  |  |  | 1.9 |
|  | 791 Z=2^a^ | 8GalA+4Me~☆ |  |  | 2.9 | 3.1 |
|  | 798 Z=2^a^ | 8GalA+5Me~☆ |  |  |  | 1.5 |
| DP9 | 879 Z=2^a^ | 9GalA+4Me~☆ |  |  |  | 1.7 |
|  | 886 Z=2^a^ | 9GalA+5Me~☆ |  |  |  | 2.4 |

a [M-H]^-2^ anions b fluorescent label 2 AB

**Supplementary Table 3** Descriptive parameters for the methyl-esterified distribution of 20 HG pectins

| Class | Fraction | DM (%) | DB (%) | DB_abs_ (%) | DP_HG_ | N_oligo_ | AVE DP_unesterified_ | N_block_ | | | | |
| --- | --- | --- | --- | --- | --- | --- | --- | --- | --- | --- | --- | --- |
|  |  |  |  |  |  |  |  | Mono^a^ | Dis | Tri | Tetra | Penta |
| Ⅰ | HG-AC | 5.0 | 90.8 | 88.0 | 193 | 81 | 18 | 7 | 2 | 0 | 0 | 0 |
|  | HG-PN-2 | 6.6 | 86.3 | 80.5 | 80 | 31 | 15 | 3 | 1 | 0 | 0 | 0 |
|  | HG-PJ-2 | 9.6 | 76.2 | 68.0 | 96 | 37 | 10 | 6 | 2 | 0 | 0 | 0 |
| Ⅱ | HG-PN-1 | 11.2 | 74.8 | 65.9 | 79 | 20 | 12 | 3 | 1 | 0 | 0 | 0 |
|  | HG-PJ-1 | 17.6 | 68.9 | 56.8 | 66 | 24 | 8 | 3 | 2 | 1 | 0 | 0 |
|  | HG-PV-2 | 19.8 | 68.9 | 59.0 | 93 | 34 | 9 | 5 | 2 | 1 | 0 | 0 |
| Ⅲ | HG-PH-2 | 21.8 | 57.4 | 46.8 | 120 | 42 | 8 | 8 | 3 | 1 | 0 | 0 |
|  | HG-AA-2 | 23.5 | 65.7 | 55.7 | 66 | 24 | 7 | 4 | 2 | 1 | 0 | 0 |
|  | HG-SF | 23.7 | 55.2 | 43.0 | 183 | 61 | 7 | 11 | 6 | 4 | 0 | 0 |
|  | HG-PH-1 | 25.0 | 59.9 | 48.8 | 86 | 29 | 7 | 5 | 3 | 1 | 0 | 0 |
|  | HG-AA-1 | 25.2 | 59.6 | 48.4 | 47 | 17 | 6 | 4 | 2 | 0 | 0 | 0 |
|  | HG-PO-1 | 27.2 | 60.6 | 48.0 | 116 | 43 | 6 | 7 | 4 | 2 | 1 | 0 |
|  | HG-PO-2 | 27.7 | 60.7 | 48.6 | 97 | 34 | 6 | 6 | 3 | 2 | 1 | 0 |
|  | HG-CC | 28.9 | 47.5 | 34.9 | 65 | 20 | 5 | 4 | 2 | 2 | 1 | 0 |
| Ⅳ | HG-PV-1 | 30.3 | 53.6 | 41.1 | 54 | 17 | 5 | 4 | 2 | 2 | 0 | 0 |
|  | HG-KL-2 | 31.0 | 58.4 | 46.2 | 116 | 43 | 6 | 8 | 4 | 2 | 1 | 0 |
|  | HG-SC-2 | 33.5 | 45 | 32.1 | 58 | 18 | 4 | 4 | 2 | 2 | 1 | 0 |
|  | HG-SC-1 | 34.8 | 33.6 | 22.0 | 260 | 66 | 5 | 13 | 10 | 8 | 4 | 2 |
|  | HG-II | 39.0 | 38.2 | 25.6 | 146 | 47 | 5 | 8 | 5 | 4 | 2 | 1 |
|  | HG-KL-1 | 40.2 | 25.5 | 15.9 | 90 | 21 | 4 | 5 | 3 | 3 | 2 | 1 |

a: Mono stands for mono-esterified block; Dis stands for di-esterified block; Tri stands for tri-esterified block; Tetra stands for tetra-esterified block; Penta stands for penta-esterified block
